# Prioritization of Genetic Variants in the microRNA Regulome as Functional Candidates in Genome-Wide Association Studies

**DOI:** 10.1002/humu.22337

**Published:** 2013-05-08

**Authors:** Brendan Bulik-Sullivan, Sara Selitsky, Praveen Sethupathy

**Affiliations:** 1 Department of Genetics, University of North Carolina at Chapel HillChapel Hill, North Carolina; 2 Carolina Center for Genome Sciences, University of North Carolina at Chapel HillChapel Hill, North Carolina; 3 Lineberger Comprehensive Cancer Center, University of North Carolina at Chapel HillChapel Hill, North Carolina

**Keywords:** microRNA, GWAS, gene regulation, polymorphism, complex disease

## Abstract

Comprehensive analyses of results from genome-wide association studies (GWAS) have demonstrated that complex disease/trait-associated loci are enriched in gene regulatory regions of the genome. The search for causal regulatory variation has focused primarily on transcriptional elements, such as promoters and enhancers. microRNAs (miRNAs) are now widely appreciated as critical posttranscriptional regulators of gene expression and are thought to impart stability to biological systems. Naturally occurring genetic variation in the miRNA regulome is likely an important contributor to phenotypic variation in the human population. However, the extent to which polymorphic miRNA-mediated gene regulation underlies GWAS signals remains unclear. In this study, we have developed the most comprehensive bioinformatic analysis pipeline to date for cataloging and prioritizing variants in the miRNA regulome as functional candidates in GWAS. We highlight specific findings, including a variant in the promoter of the miRNA let-7 that may contribute to human height variation. We also provide a discussion of how our approach can be expanded in the future. Overall, we believe that the results of this study will be valuable for researchers interested in determining whether GWAS signals implicate the miRNA regulome in their disease/trait of interest.

## Introduction

In the past 6 years, genome-wide association studies (GWAS) have identified over 7,000 unique genetic loci associated with hundreds of complex traits and diseases [Hindorff et al., 2009]. Comprehensive analyses of these findings have found that GWAS signals are significantly overrepresented in regulatory regions of the genome [Ernst et al., 2011; Hindorff et al., 2009; Maurano et al., 2012; Nica et al., 2010; Nicolae et al., 2010; Schaub et al., 2012]. Moreover, several follow-up functional studies have identified specific regulatory elements that underlie genetic associations and are directly implicated in complex disease etiology [Gaulton et al., 2010; Harismendy et al., 2011; Musunuru et al., 2010; Pomerantz et al., 2009; Stitzel et al., 2010]. Although much of the focus on regulatory variation has been centered on transcriptional elements, such as promoters and long-range enhancers [Degner et al., 2012], a seminal study from the Georges lab in 2006 found the first example of a variant in a microRNA (miRNA) target site contributing to a disease phenotype (muscular hypertrophy in Texel sheep) [Clop et al., 2006]. Five years later, Brest et al. (2011) identified the very first case of a human GWAS signal that may be explained by polymorphic miRNA targeting—a synonymous variant that alters a miR-196 target site and influences risk for Crohn’s disease [Georges 2011]. Following that report, another group implicated the miR-137 locus in schizophrenia (2011), though the molecular mechanism underlying the association remains unclear. Most recently, in 2012, Richardson et al. (2012) demonstrated that a variant in the 3′-untranslated region (3′-UTR) of *lipoprotein lipase* (*LPL*) disrupts the binding of miR-410 and modulates the effect of diet on plasma lipid levels. These findings highlight the importance of expanding the view of the regulatory variation landscape beyond chromatin and transcriptional control elements.

miRNAs are short (∼22 nucleotide) noncoding RNAs that regulate gene expression principally at the posttranscriptional level [Bartel 2009]. The human genome encodes for over 1,000 miRNAs in either intragenic regions or independent transcription units [Griffiths-Jones et al., 2008; Kozomara and Griffiths-Jones 2011]. miRNA loci are transcribed predominantly by RNA Polymerase II [Cai et al., 2004; Lee et al., 2004], yielding primary transcripts (pri-miRNAs) of highly variable length depending on the locus [Saini et al., 2008; Stitzel et al., 2010]. The pri-miRNA is processed within the nucleus by the ribonuclease Drosha and its cofactors, generating one or more precursor sequences (pre-miRNAs) with a hairpin-like secondary structure [Kim 2005; Kim et al., 2009]. The pre-miRNA is then exported to the cytoplasm, where it is subject to further enzymatic processing by Dicer and its partners, producing a ∼22 bp double stranded RNA duplex. One strand of this duplex, referred to as the mature miRNA, is loaded onto the RNA-induced silencing complex (RISC). The miRNA guides and tethers the RISC to specific target RNAs to regulate their stability and/or translation [Bartel 2009].

The miRNA regulome (defined as the compendium of regulatory elements that either regulate miRNA expression or are regulated by miRNA activity) is a critical component of the biological networks that govern cellular and systemic phenotypes [Gennarino et al., 2012; Liang and Landweber 2007; Volinia et al., 2010]. miRNAs have emerged as stable plasma biomarkers for disease diagnosis and prognosis [Mitchell et al., 2008], and as promising therapeutic targets for a growing number of disorders [Jackson and Levin 2012; van Rooij et al., 2012]. Recent studies of genetic variation in human populations demonstrated that purifying selection has constrained the genetic diversity of the miRNA Regulome [Chen and Rajewsky 2006; Hu and Bruno 2011; Li et al., 2012a; Quach et al., 2009; Saunders et al., 2007]. In fact, Chen and Rajewsky reported in their seminal 2006 study that negative selection may be stronger on predicted conserved miRNA target sites than on most other functional classes of genomic elements, including nonsynonymous sites [Chen and Rajewsky 2006]. These findings suggest that genetic variation in the miRNA regulome may have strongly deleterious phenotypic consequences. Notably, however, several of the same studies also identified islands of the miRNA regulome that have been subject to recent positive selection [Li et al., 2012a; Liang and Li 2009; Quach et al., 2009; Saunders et al., 2007]. Taken together, these observations indicate that genetic variation in the miRNA regulome contributes to both population adaptation and complex disease etiology.

Human genetic variation in the miRNA regulome, particularly miRNA target sites, has been extensively catalogued [Barenboim et al., 2010; Bhattacharya et al., 2012; Gong et al., 2012; Hariharan et al., 2009; Hiard et al., 2010; Landi et al., 2011; Schmeier et al., 2011; Zorc et al., 2012], and has been demonstrated to have widespread effects on miRNA-mediated gene regulation [Gamazon et al., 2012; Kim and Bartel 2009; Lu and Clark 2012]. Although the extent to which polymorphic miRNA-mediated gene regulation contributes to phenotypic variation is not clear [Sethupathy and Collins 2008], it is an area that could have broad implications for disease biology [Borel and Antonarakis 2008; Chen et al., 2008; Gamazon et al., 2012; Georges et al., 2007] and pharmacogenomics [Mishra et al., 2008] and as such, clearly merits further investigation.

Over the last 2 years, several bioinformatic strategies and web servers have been developed to facilitate the identification of validated GWAS signals that alter the miRNA regulome [Arnold et al., 2012; Bruno et al., 2012; Li et al., 2012b; Richardson et al., 2011; Thomas et al., 2011; Ziebarth et al., 2012]. However, these approaches harbor several major limitations that hinder the effective prioritization of variants for functional validation. In this study, we present the most comprehensive strategy to date that addresses each of these limitations. We highlight specific findings and also discuss how the approach can be expanded in the future. We believe that the results of this study will be very valuable for researchers interested in determining whether GWAS signals implicate the miRNA regulome in their disease/trait of interest.

## Materials and Methods

### Defining Linkage Disequilibrium Blocks of Trait/Disease Association

The NHGRI GWAS catalog (as of 11/14/12) was mined for all single nucleotide polymorphisms (SNPs) reported to be associated (*P* < 1.0 × 10^−5^) with a trait/disease. For simplicity sake, multi-SNP haplotypes (*n* = 46) were not considered. For each study, the following information was recorded: first author, row number in GWAS catalog, PubMed ID, index SNP ID, trait/disease, case–control cohort ancestry, and association *P* value.

Each GWAS was assigned to one of four super-populations in the 1000 Genomes Project (1000G) according to the mapping scheme described in Box 1. For each index SNP reported in each GWAS, 1000G SNPs in linkage disequilibrium (LD) (defined as *r*^2^ > 0.6) were identified by mining the 1000G phase I haplotype data for the assigned super-population (http://www.sph.umich.edu/csg/yli/mach/download/1000G.2012-02-14.html). For every SNP in an LD block of association, the following information from 1000G was recorded: chromosomal location (hg19), extent of LD with index SNP (*r*^2^), and minor allele identity/frequency in the assigned super-population.

**BOX 1. GWAS Assignments to One of Four Super-Populations of the 1000 Genomes Project**

**Table:** 

Case–control cohort ancestry information provided by the GWAS	1000G super-population assignment
Indian|India|Europe|Canadian|Canada|Caucasian|White|Framingham|Norwegian|Norway|French|France|Iceland|German|Germany|Scandanavia|Finnish|Finland| UK |Welsh|Wales|Irish|Ireland|Scottish|Scotland|Spanish|Spain|Iberia|Toscani|Tuscan|Utah|CEPH|England|English|Swiss|Switzerland|Australia|Turkish|Turkey|Saami|Belgium|Belgian|Russia|Polish|Poland	EUR (European)
Africa|Ghani|Malawi|Yoruba|Esan|Mende|Sierra|Leone|Gambia|Kenya|Luhyai	AFR (African)
Mexico|Mexican|Hispanic|Peru|Puerto|Rico|Rican|Colombia|Medellin|Lima	AMR (American)
Asia|Chin|Japan|Thai|Korea|Bangladesh|Taiwan|Indonesia|Vietnam|Hong Kong|Kosrae|Micronesia|Papua New Guinea|Han|Dai|Kinh |Tokyo|Xishuangbanna|Beijing	ASN (Asian)
Anything else	EUR (European)

### miRNA Regulome Datasets

Human miRNA and pre-miRNA locations were downloaded from miRBase version 18 (http://www.mirbase.org/). Promoter regions of human miRNAs were obtained from epigenomic studies in two primary human cell types: CD4+ T cells [Barski et al., 2009] and pancreatic islets [Stitzel et al., 2010]. 3′-UTR sequences from the reference genomes of human, mouse, rat, dog, and chicken were downloaded from TargetScan 6.1 (http://www.targetscan.org/cgi-bin/targetscan/data_download.cgi?db=vert_61). Coordinates for the human 3′-UTR sequences were obtained by running a command line version of BLAT against the human genome (hg19). 3′-UTRs that mapped perfectly to multiple locations, or that appeared to be spliced, or were of length <20 nt (BLAT requires sequences >20 nt) were discarded from the analysis. For each 3′-UTR, the RefSeq ID provided by TargetScan 6.1 was converted to the official gene symbol using the BioMart database (http://useast.ensembl.org/biomart/martview/).

Human miRNA target sites were predicted within the reference 3′-UTR sequences using the TargetScanS algorithm (written in Perl and executed on a local server). For each prediction, the following information was recorded: miRNA name, gene symbol, target site type (7mer-1a, 7mer-m8, and 8mer-1a, in order of increasing efficacy), and a conservation number (1–5, indicating the number of species among [human, mouse, rat, dog, chicken] in which the putative target site is exactly conserved; a value of 1 indicates that the site is present only in the human and not in any of the other four species analyzed).

For each human 3′-UTR reference sequence, the allelic complement was generated by replacing every reference allele with the nonreference allele at each bi-allelic polymorphic locus reported by 1000G. If two or more SNPs are within the span of a target site (7 nt), all combinations of reference and alternate alleles at these sites were considered (e.g., if two A/T SNPs are within 7 nt, each of the potential AT, TA, and TT haplotypes would be considered). Many SNPs in close proximity are likely to be in strong LD; therefore, some of these allelic combinations are likely very rare. However, they have been included for comprehensiveness. Human miRNA target sites were predicted within the allelic complement 3′-UTR sequences using the TargetScanS algorithm. For each prediction, the same information as for predictions in the reference 3′-UTRs was recorded.

### Other Functional Annotation Datasets

- Coding exons were downloaded from the “knownGenes” table on the UCSC Table Browser (https://genome.ucsc.edu/cgi-bin/hgTables).
- Nonsynonymous SNPs (nonsense, missense, frameshift) were downloaded from dbSNP build 137 (ftp://ftp.ncbi.nih.gov/snp/organisms/human_9606/VCF/00-All.vcf.gz).
- Transcriptional SNPs were downloaded from RegulomeDB (http://regulome.stanford.edu/downloads).
- Expression quantitative trait loci (eQTLs) in lymphocytes were obtained from (P.F. Sullivan, unpublished data).
- Validated miRNA:target-gene pairs were downloaded from TarBase 5.0 (http://diana.cslab.ece.ntua.gr/tarbase/tarbase_download.php).

### Identification of Candidate miRNA Regulatory Hubs

Candidate gene lists for each trait/disease were extracted from the NHGRI GWAS catalog. For each trait/disease, potential miRNA regulatory hubs in the underlying gene network were identified by Monte Carlo simulation analysis. First, the seed-based TargetScanS algorithm was used to determine the number of predicted conserved targets in the gene list for each miRNA. This number was converted to a score by weighting 8-mer seed matches or target sites within 60 nt by a factor of 1.5. This procedure was repeated 100,000 times with a new set of randomly selected genes from the human genome each time, to generate a background expectation of the targeting score for each miRNA, which was then used to calculate an empirical *P* value for the score obtained with the candidate gene list for the trait/disease. To account for differences in the average 3′-UTR length between the trait/disease genes of interest and the randomly selected genes in each simulation, the number of predicted target genes was normalized to the average 3′-UTR length in the following manner. Specifically, the following equation is used: 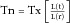, where 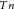 is the normalized number of predicted target genes in a random simulation, *T* is the actual number of predicted target genes in a random simulation, 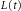 is the average length of the 3′-UTRs in the test set, and 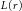 is the average length of the 3′-UTRs in the random set.

## Results

### Strategy

We developed an integrative genomic pipeline to catalog and prioritize trait/disease-associated single nucleotide polymorphisms (TASs) in the miRNA regulome. TASs include SNPs reported by GWAS (index SNPs) and all other SNPs in strong LD. We describe below five features of our strategy that represent conceptual and/or empirical advances relative to the existing approaches:

1. *Inclusion of nonconserved miRNA target sites*. Current approaches have largely restricted their analyses to predicted miRNA target sites that are highly conserved. However, recent integrative genomic analyses indicate that GWAS loci are enriched in nonconserved regions of the genome [Gaffney et al., 2012; Schaub et al., 2012]. Therefore, it is likely that overlooking lineage-specific miRNA target sites misses many causative variants. We have included in our pipeline any miRNA target site that is predicted in the human genome regardless of the extent of cross-species conservation (Methods).
2. *Definition of LD blocks of association using data from the 1000 Genomes Project*. Current approaches compute LD using genotype data from the International HapMap project [Altshuler et al., 2012]. Instead, we have chosen to define LD blocks based on the sequence data from phase I of the 1000 Genomes Project (1000G) (Methods), because it provides the highest resolution human genetic map to date [Abecasis et al., 2012]. Specifically, compared with HapMap, the 1000G resource doubles the number of variants that are in LD with each GWAS index SNP [Abecasis et al., 2012].
3. *Definition of population-specific LD blocks of association*. Current approaches consider only the LD structure in HapMap individuals of European descent. To account for the increasing number of GWAS in non-European populations and the varying LD patterns across different populations, we have defined LD blocks for each index SNP from each GWAS using the 1000G data for the “super population” (African, Asian, American, European) that most closely matches the ancestry of the case-control cohort used in the GWAS (Methods).
4. *Inclusion of miRNA promoter regions*. Until recently, promoters of miRNAs were largely unknown and were not considered in most surveys of genetic variation in the miRNA regulome. In last few years, several groups, including our own, have used large-scale epigenomic strategies to annotate comprehensively the promoter regions of miRNAs [Barski et al., 2009; Corcoran et al., 2009; Marson et al., 2008; Ozsolak et al., 2008; Stitzel et al., 2010]. Given that genetic variants in miRNA promoters could alter miRNA expression and function [Luo et al., 2011], and that gene promoters are enriched for GWAS loci [Hindorff et al., 2009], we have incorporated miRNA promoter annotations from two different studies into the analysis pipeline (Methods).
5. *Functional annotation of all SNPs within LD association blocks to assess the likelihood that the association signal is explained by the miRNA regulome*. Current approaches do not assess whether the association signal is likely to be explained by a gene regulatory mechanism, and if it is, whether any of the genetic variants in the associated LD block may have other (non-miRNA-related) compelling regulatory annotations. Therefore, for every TAS in the miRNA regulome, we annotate whether any of the SNPs in the corresponding GWAS LD block occur at exons, nonsynonymous sites, and/or transcriptional regulatory elements as defined by RegulomeDB [Boyle et al., 2012], which includes all of the high-throughput experimental datasets generated by the Encyclopedia of DNA Elements (ENCODE) Project[Dunham et al., 2012]. TASs in LD blocks uniquely associated with the miRNA regulome (i.e., LD blocks that do not harbor any other exonic or transcriptional SNP) are deemed to be of highest priority for functional validation. Finally, for every candidate miRNA:trait pair that we identify, we perform a Monte Carlo simulation to determine whether the miRNA is a candidate regulatory hub in the network of genes implicated in the trait by GWAS.

### Trait/Disease-Associated Genetic Variants in the miRNA Regulome

**Figure 1:**
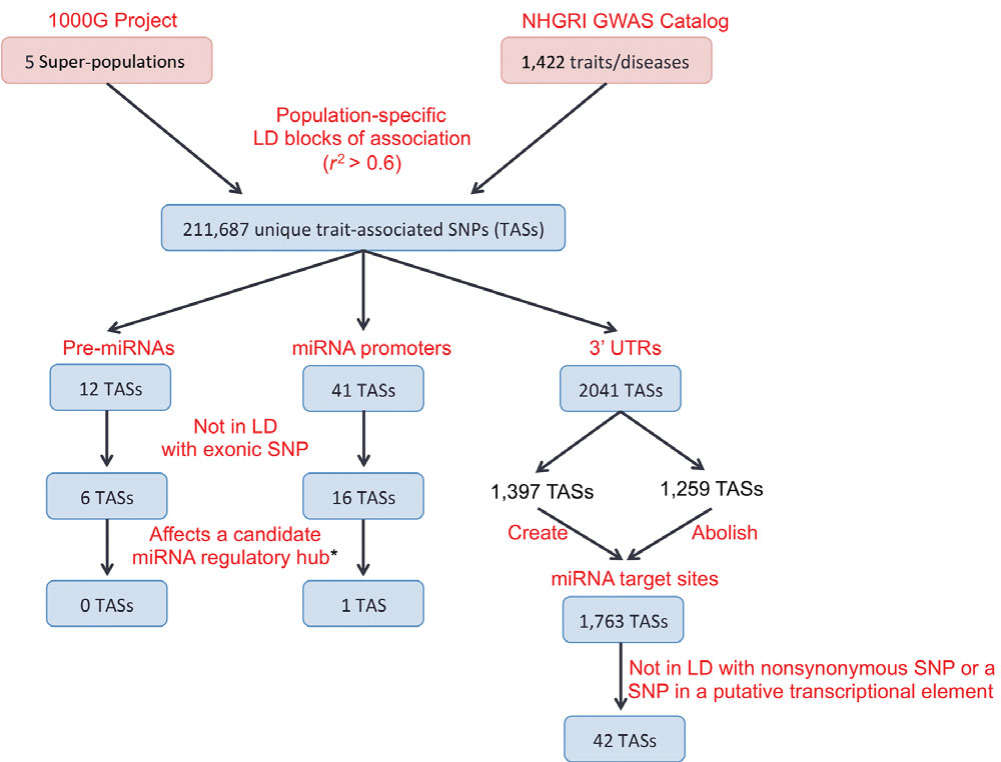
Summary of the analysis pipeline. The primary data sources (pink rectangles) and the number of trait-associated SNPs (blue rectangles) passing each filter (red text) in the analysis pipeline are shown. ^*^, candidate miRNA regulatory hubs are identified for each trait/disease using a Monte Carlo simulation strategy (Methods).

Our integrative analysis of the NHGRI GWAS catalog and the 1000G database identified 211,687 unique TASs (Fig. 1). Of these, 12, 41, and 2,041 TASs occur within miRNA precursors, miRNA promoter regions, and 3′-UTRs, respectively.

#### miRNA precursors

The low density of TASs in pre-miRNAs is consistent with previous reports and is suggestive of negative selection on miRNA loci. Of the 12 TASs within pre-miRNAs, six are in GWAS LD blocks that do not contain any known exonic variant (Fig. 1). Among these six is rs12803915 (Table 1; Supp. Table S1), which is located within the precursor of a primate-specific miRNA (miR-612), and is in moderate LD (1000G EUR, *r*^2^ = 0.6) with a reported index SNP (rs17146964) for vertical cup-disc ratio (CDR; a parameter linked to glaucoma risk) in a case-control cohort of European ancestry. A very recent study demonstrated in several cell lines that the minor allele of rs12803915 significantly alters the cellular processing of pre-miR-612 and, consequently, the expression levels of mature miR-612 [Kim et al., 2012]. This pre-miRNA TAS is a compelling candidate for further validation as a determinant of CDR.

**Table 1:** Selected Examples of Trait/Disease-Associated SNPs (TASs) in the miRNA Regulome

Trait/disorder	miRNA regulome SNP	Minor allele frequency	miRNA	miRNA regulome element
Vertical cup-disc ratio	rs12803915	21.4% (EUR)	miR-612	Pre-miRNA
Pulse rate	rs6701558	11.2% (ASN)	miR-29b/c	Promoter region
Schizophrenia	rs2660302	21.8% (EUR)	miR-137	Promoter region
Height	rs113431232	3.4% (EUR)	let-7a/d/f	Promoter region
Pigmentation	rs35407	2.2% (EUR)	miR-27	*SLC45A2* target site
Body mass index	rs77632545	19.9% (ASN)	miR-181a	*ZNF169* target site
Asthma	rs17052784	2.6% (EUR)	miR-140-3p	*DCLK1* target site
Plasma C-reactive protein levels	rs116971887	4.9% (EUR)	miR-194	*SALL1* target site
Type 1 diabetes autoantibodies	rs3842753	50.0% (EUR)	miR-491-5p	*INS* target site
Type 2 diabetes	rs1802295	50.0% (ASN)	miR-510	*VPS26*A target site

Of the 42 TASs that are in the miRNA regulome and are not in LD with annotated nonsynonymous or transcriptional SNPs, 10 are shown here (pre-miRNA, *n* = 1; miRNA promoter, *n* = 3; miRNA target site, *n* = 6). Minor allele frequencies (MAFs) are specific to the 1000G super-population (ASN, Asian; EUR, European; AMR, American; AFR, African) that is closest to the ancestry of the case-control cohort in the GWAS that identified the genetic association. MAFs of the miRNA regulome SNPs range from relatively rare (e.g., rs35407, EUR MAF = 0.022) to very common (e.g., rs1802295, ASN MAF = 0.5).

#### miRNA promoter regions

Of the 41 TASs within miRNA promoters, 16 are in GWAS LD blocks that do not contain any known exonic variant (Fig. 1). Among these 16 is rs6701558 (Table 1; Supp. Table S1), which is within the promoter of the miR-29b-2/miR-29c cluster, and is in complete LD (1000G ASN, *r*^2^ = 1) with a reported index SNP (rs12731740) for pulse rate in a cohort of Asian ancestry. This finding is consistent with the recent observation that the miR-29 family of miRNAs control aortic dilation and aneurysm formation [Boon et al., 2011], which directly influence heart rate. Another compelling candidate for functional evaluation is rs2660302 (Table 1; Supp. Table S1), which occurs within the promoter of miR-137, and is in LD (1000G EUR, *r*^2^ = 0.71) with an index SNP (rs1625579) for schizophrenia in a cohort of European ancestry. Two previous studies demonstrated that miR-137 is a candidate regulatory hub in the schizophrenia gene network [2011; Potkin et al., 2010]. The rs2660302 locus may mediate allele-dependent transcriptional regulation of miR-137, thereby altering the expression status of genes underlying the etiology of schizophrenia.

**Figure 2:**
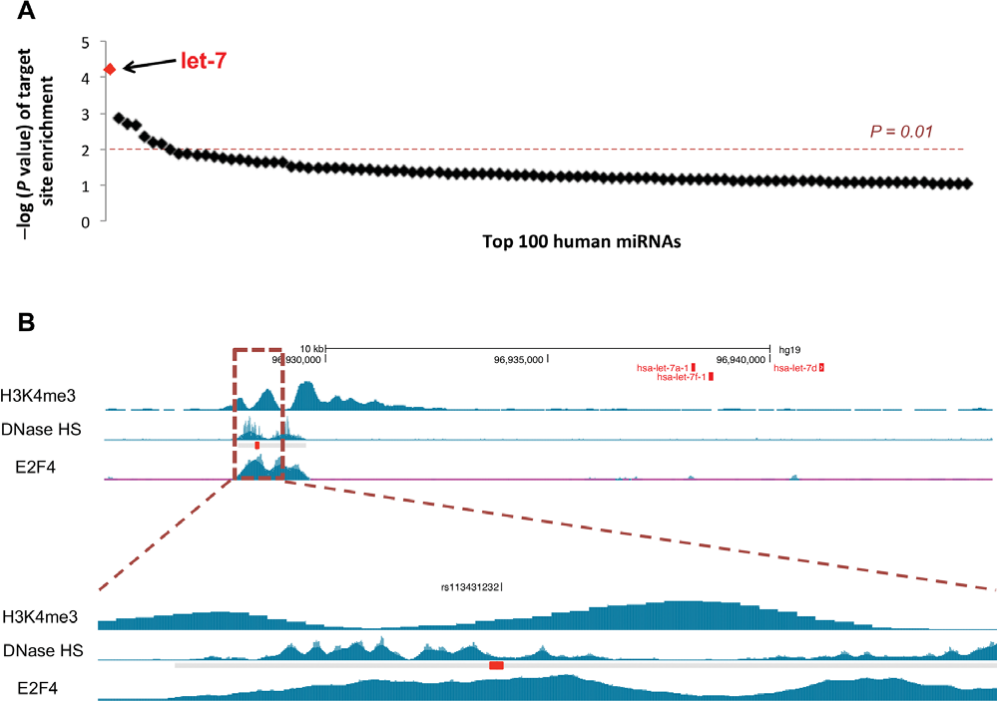
A genetic variant in the let-7 promoter may contribute to human height variation. A: Each data point represents a human miRNA and the y-axis shows the log10 of the *P* value of miRNA target site enrichment among genes implicated in height by GWAS. The dashed line denotes the significance threshold (empirical *P* = 0.01). B: SNP rs113431232, which is in LD with an index SNP (rs1257763) for height, occurs in a validated E2F4 binding site within the promoter region of the let-7a/d/f miRNA cluster. H3K4me3 (histone H3 lysine 4 tri-methylation) ChIP-seq signal (ENCODE) denotes promoter regions; DNase HS (DNase I hypersensitive site) signal (ENCODE) denotes open chromatin; E2F4 ChIP-seq signal (ENCODE) denotes chromatin occupancy of transcription factor E2F4. Peak E2F4 occupancy signal (red rectangle) occurs within a local dip in the DNase HS signal, which is indicative of a bound transcription factor(s). All ENCODE data shown are from HeLa cells.

To identify the most compelling functional candidates among the remaining 15 miRNA promoter TASs, we implemented a Monte Carlo simulation strategy to determine for each of the 15 miRNA:trait pairs whether the miRNA is predicted to target significantly more of the protein-coding genes implicated in the trait by GWAS than expected by chance. The most striking result was for the pair let-7:height (Fig. 2A). Specifically, we found that predicted target sites for let-7 are the most significantly overrepresented within the 3′-UTRs of genes at loci associated with human stature (Fig. 2A). The TAS (rs113431232) located within the let-7 promoter region has a minor allele frequency (MAF) of ∼3% in the 1000G population of European ancestry and is in LD (1000G EUR, *r*^2^ = 0.74) with a reported index SNP (rs1257763) for height (Table 1; Supp. Table S1). We mined the ENCODE database and determined that this TAS occurs within a region of open chromatin in many cell types (Fig. 2B), highlighting the possibility that it may influence the transcriptional activity of the locus. We then analyzed the ENCODE data generated by chromatin immunoprecipitation followed by high-throughput sequencing (ChIP-seq) and found that the TAS overlaps a high-confidence binding site in HeLa cells for the transcription factor E2F4 (Fig. 2B). Notably, a recent study validated this binding site by ChIP-PCR and also demonstrated that overexpression of E2F4 in HeLa cells leads to down-regulation of let-7 [Lee et al., 2011]. This finding is consistent with the known oncogenic and tumor suppressive capacity of E2F4 and let-7, respectively [Beijersbergen et al., 1994; Roush and Slack 2008]. The minor allele at rs113431232 may disrupt E2F4 binding, thereby upregulating let-7 and altering the expression of targets that influence cell growth and, ultimately, human stature.

#### miRNA target sites

Of the 2,041 TASs located in 3′-UTRs, 1,763 create and/or abolish predicted target sites for human miRNAs (Fig. 1; Supp. Table S2). One of these, rs13702, has recently been validated by rigorous follow-up genetic and molecular studies [Richardson et al., 2012]. Specifically, Richardson et al. (2012) demonstrated that the rs13702 minor allele reduces plasma lipid levels by abolishing a miR-410 target site in the 3′-UTR of *LPL*, which leads to increased LPL expression and activity. Two other TASs, rs12537 and rs12904, which are associated with IgA nephropathy and gamma-glutamyl transferase (GGT) levels, respectively, have been implicated previously in gastric cancer by candidate gene association studies. *In vitro* molecular experiments have confirmed that the rs12537 minor allele creates a miR-181a target site in *MTMR3* [Lin et al., 2012] and that the rs12904 minor allele disrupts a highly conserved miR-200bc target site in *EFNA1* [Li et al., 2012c]. However, neither has yet been verified as the mechanistic link for the genetic association with IgA nephropathy and GGT levels, respectively. Although both rs12537 and rs12904 are in strong LD with nonsynonymous variants and SNPs in predicted transcriptional regulatory elements (Supp. Table S2), the miRNA-related mechanism may merit further investigation. We performed an analysis of TarBase 5.0, a repository of experimentally supported miRNA:target-gene pairs (Methods), and identified one additional TAS (rs891368) that occurs within a predicted miRNA target site for a gene (*RFT1*) that has been previously demonstrated to be regulated by the same miRNA (miR-15). rs891368 is in perfect LD (*r*^2^ = 1) with a reported index SNP (rs2336725) for height (Supp. Table S2).

Among the 1,763 TASs within miRNA target sites, 42 are in GWAS LD blocks that do not contain either a nonsynonymous or a transcriptional regulatory variant (Fig. 1; Supp. Table S2). Of these, 26 are either in very strong LD (*r*^2^ > 0.9) with the reported index SNPs or are the index SNPs themselves. Several such TASs are highlighted in Table 1, two of which are described in further detail below.

The minor allele at rs116971887 disrupts a highly conserved predicted miR-194 target site within the gene *SALL1*, and is in strong LD (1000G EUR, *r*^2^ = 0.92) with a reported index SNP for plasma C-reactive protein (CRP) levels (Table 1; Supp. Table S2), which is a strong indicator of cardio-metabolic status [Onat et al., 2008]. SALL1 is critical for normal development and function of the kidney [Abedin et al., 2011; Nishinakamura and Takasato 2005], where miR-194 expression is highly enriched [Senanayake et al., 2012]. CRP is produced in the kidney in response to inflammatory signals [Jabs et al., 2003] and is associated with abnormalities in kidney function [Stuveling et al., 2003]. The minor allele at rs116971887 is predicted to abrogate the miR-194 target site, thereby potentially increasing SALL1 expression levels. The question of whether this would lead to increased plasma CRP merits further detailed experimentation.

The minor allele at rs77632545 is predicted to create a new target site for miR-181a within the 3′-UTR of *ZNF169*, and is in strong LD (1000G ASN, *r*^2^ = 0.95) with a reported index SNP for body mass index (BMI). *ZNF169* is a member of the zinc-finger family of genes, which have been identified as harboring unusually effective coding region target sites specifically for miR-181a [Huang et al., 2010; Schnall-Levin et al., 2011]. It has also been demonstrated that when miRNAs bind to messenger RNAs in both the coding region and the 3′-UTR the repressive effect on protein expression is synergistic [Fang and Rajewsky 2011]. Therefore, by creating a new miR-181a target site, the minor allele at rs77632545 could induce significantly decreased levels of ZNF169. Further investigation is required to determine whether altered expression of ZNF169 is associated with, or can directly influence, BMI and related phenotypes.

## Discussion

We have developed the most comprehensive approach to date for cataloging trait/disease-associated genetic variation in the miRNA regulome. Our analysis pipeline provides a concrete means of prioritizing variants in the miRNA regulome as functional candidates in GWAS. We described here specific miRNA-related variants that may explain genetic associations with a variety of traits/diseases, including body mass index, IgA nephropathy, and schizophrenia.

Our study is the first to catalog systematically trait/disease-associated genetic variants in miRNA promoter regions. These promoters were obtained from epigenomic analyses conducted in two different human cell types [Barski et al., 2009; Stitzel et al., 2010]. In future genetic analyses of the miRNA regulome, it will be meaningful to include a more comprehensive set of miRNA promoters, which can be identified by analyzing relevant chromatin data that have been generated by the ENCODE and NIH Epigenomics Roadmap consortiums for a wide array of different cell types and physiologic conditions. Furthermore, the application of chromosome conformation capture technology may facilitate the identification of long-range regulatory elements (e.g., enhancers and silencers) that contribute to the control of miRNA expression, and may also harbor trait/disease-causing genetic variants.

In our survey, we included the broadest set to date of predicted miRNA target sites based on an algorithm that identifies sequences within 3′-UTRs that are exactly complementary to the 5′-end of a miRNA, referred to as the “seed” region [Friedman et al., 2009]. It has been demonstrated that some miRNAs have alternative modes of target recognition that do not require a seed match [Brennecke et al., 2005; Chi, et al., 2012; Shin et al., 2010]. Also, miRNAs can effectively target regions outside the 3′-UTR, including the open reading frame [Forman and Coller 2010]. Incorporating these categories of target sites will expand the functional miRNA regulome and facilitate the identification of trait/disease associated genetic variants that disrupt miRNA activity.

It is worth noting that variants beyond the miRNA regulome can still affect miRNA activity. For example, it has been demonstrated that 3′-UTR variants outside of a miRNA target site can influence miRNA targeting efficacy [Mishra et al., 2007], potentially by altering local secondary structure and accessibility to the miRNA-RISC [Haas et al., 2012]. It is well established that genetic variants can alter mRNA folding [Shen et al., 1999], which in turn can influence function [Halvorsen et al., 2010]. Very recently, high-throughput approaches have been developed to resolve RNA structures [Deigan et al., 2009; Lucks et al., 2011] toward the goal of predicting the effect of specific variants on secondary and tertiary RNA conformation [Ritz et al., 2012]. As these strategies become increasingly tractable, it will be of great interest to assess the extent to which trait/disease-associated genetic variants that are not explicitly within the miRNA regulome nevertheless disrupt miRNA activity.

In summary, our strategy improves upon the accuracy and resolution of previous approaches and, importantly, facilitates the prioritization of genetic variants in the miRNA regulome as functional candidates in GWAS. Although we do not provide experimental validation in this study, we have highlighted specific examples that merit further detailed investigation, including a SNP associated with height that occurs within a validated E2F4 binding site in the let-7 promoter, and a SNP associated with vertical CDR that has been shown to alter miR-612 biogenesis. The bioinformatic pipeline presented in this study can be extended in the future to incorporate many other types of genomic data, such as miRNA expression patterns and somatic mutations in cancer.

Complex diseases are increasingly viewed as “network disorders” [Sullivan 2012]. Biological networks often have mechanisms for conferring robustness against genetic and/or environmental perturbation, in part due to a web of miRNA activity [Ebert and Sharp 2012]. Therefore, genetic variants that alter miRNA activity will likely have a dramatic effect on network output. We expect that as more high-powered genetic association studies are performed, and as the functional miRNA regulome is brought into clearer view, an increasing number of miRNA-related variants will be implicated in complex disease etiology.

## Notes

Disclaimer: Supplementary materials have been peer-reviewed but not copyedited.

## References

[b1] Schizophrenia Psychiatric Genome-Wide Association Study (GWAS) Consortium (2011). Genome-wide association study identifies five new schizophrenia loci. Nat Genet.

[b2] Abecasis GR, Auton A, Brooks LD, DePristo MA, Durbin RM, Handsaker RE, Kang HM, Marth GT, McVean GA (2012). An integrated map of genetic variation from 1,092 human genomes. Nature.

[b3] Abedin MJ, Imai N, Rosenberg ME, Gupta S (2011). Identification and characterization of Sall1-expressing cells present in the adult mouse kidney. Nephron. Exp Nephrol.

[b4] Altshuler DM, Gibbs RA, Peltonen L, Altshuler DM, Gibbs RA, Peltonen L, Dermitzakis E, Schaffner SF, Yu F, Peltonen L, Dermitzakis E, Bonnen PE (2010). Integrating common and rare genetic variation in diverse human populations. Nature.

[b5] Arnold M, Ellwanger DC, Hartsperger ML, Pfeufer A, Stumpflen V (2012). Cis-acting polymorphisms affect complex traits through modifications of microRNA regulation pathways. PloS ONE.

[b6] Barenboim M, Zoltick BJ, Guo Y, Weinberger DR (2010). MicroSNiPer: a web tool for prediction of SNP effects on putative microRNA targets. Hum Mutat.

[b7] Barski A, Jothi R, Cuddapah S, Cui K, Roh TY, Schones DE, Zhao K (2009). Chromatin poises miRNA- and protein-coding genes for expression. Genome Res.

[b8] Bartel DP (2009). MicroRNAs: target recognition and regulatory functions. Cell.

[b9] Beijersbergen RL, Kerkhoven RM, Zhu L, Carlee L, Voorhoeve PM, Bernards R (1994). E2F-4, a new member of the E2F gene family, has oncogenic activity and associates with p107 in vivo. GenesDev.

[b10] Bhattacharya A, Ziebarth JD, Cui Y (2012). Systematic analysis of microrna targeting impacted by small insertions and deletions in human genome. PloS ONE.

[b11] Boon RA, Seeger T, Heydt S, Fischer A, Hergenreider E, Horrevoets AJ, Vinciguerra M, Rosenthal N, Sciacca S, Pilato M, van Heijningen P, Essers J (2011). MicroRNA-29 in aortic dilation: implications for aneurysm formation. CircRes.

[b12] Borel C, Antonarakis SE (2008). Functional genetic variation of human miRNAs and phenotypic consequences. Mamm Genome.

[b13] Boyle AP, Hong EL, Hariharan M, Cheng Y, Schaub MA, Kasowski M, Karczewski KJ, Park J, Hitz BC, Weng S, Cherry JM, Snyder M (2012). Annotation of functional variation in personal genomes using RegulomeDB. Genome Res.

[b14] Brennecke J, Stark A, Russell RB, Cohen SM (2005). Principles of microRNA-target recognition. PLoS Biol.

[b15] Brest P, Lapaquette P, Souidi M, Lebrigand K, Cesaro A, Vouret-Craviari V, Mari B, Barbry P, Mosnier JF, Hebuterne X, Harel-Bellan A, Mograbi B (2011). A synonymous variant in IRGM alters a binding site for miR-196 and causes deregulation of IRGM-dependent xenophagy in Crohn’s disease. Nat Genet.

[b16] Bruno AE, Li L, Kalabus JL, Pan Y, Yu A, Hu Z (2012). miRdSNP: a database of disease-associated SNPs and microRNA target sites on 3’UTRs of human genes. BMC Genomics.

[b17] Cai X, Hagedorn CH, Cullen BR (2004). Human microRNAs are processed from capped, polyadenylated transcripts that can also function as mRNAs. RNA.

[b18] Chen K, Rajewsky N (2006). Natural selection on human microRNA binding sites inferred from SNP data. Nat Genet.

[b19] Chen K, Song F, Calin GA, Wei Q, Hao X, Zhang W (2008). Polymorphisms in microRNA targets: a gold mine for molecular epidemiology. Carcinogenesis.

[b20] Chi SW, Hannon GJ, Darnell RB (2012). An alternative mode of microRNA target recognition. Nat Struct Mol Biol.

[b21] Clop A, Marcq F, Takeda H, Pirottin D, Tordoir X, Bibe B, Bouix J, Caiment F, Elsen JM, Eychenne F, Larzul C, Laville E (2006). A mutation creating a potential illegitimate microRNA target site in the myostatin gene affects muscularity in sheep. Nat Genet.

[b22] Corcoran DL, Pandit KV, Gordon B, Bhattacharjee A, Kaminski N, Benos PV (2009). Features of mammalian microRNA promoters emerge from polymerase II chromatin immunoprecipitation data. PLoS ONE.

[b23] Degner JF, Pai AA, Pique-Regi R, Veyrieras JB, Gaffney DJ, Pickrell JK, De Leon S, Michelini K, Lewellen N, Crawford GE, Stephens M, Gilad Y, Pritchard JK (2012). DNase I sensitivity QTLs are a major determinant of human expression variation. Nature.

[b24] Deigan KE, Li TW, Mathews DH, Weeks KM (2009). Accurate SHAPE-directed RNA structure determination. Proc Natl Acad Sci USA.

[b25] Dunham I, Kundaje A, Aldred SF, Collins PJ, Davis CA, Doyle F, Epstein CB, Frietze S, Harrow J, Kaul R, Khatun J, Lajoie BR (2012). An integrated encyclopedia of DNA elements in the human genome. Nature.

[b26] Ebert MS, Sharp PA (2012). Roles for microRNAs in conferring robustness to biological processes. Cell.

[b27] Ernst J, Kheradpour P, Mikkelsen TS, Shoresh N, Ward LD, Epstein CB, Zhang X, Wang L, Issner R, Coyne M, Ku M, Durham T, Kellis M, Bernstein BE (2011). Mapping and analysis of chromatin state dynamics in nine human cell types. Nature.

[b28] Fang Z, Rajewsky N (2011). The impact of miRNA target sites in coding sequences and in 3′UTRs. PLoS ONE.

[b29] Forman JJ, Coller HA (2010). The code within the code: microRNAs target coding regions. Cell Cycle.

[b30] Friedman RC, Farh KK, Burge CB, Bartel DP (2009). Most mammalian mRNAs are conserved targets of microRNAs. Genome Res.

[b31] Gaffney DJ, Veyrieras JB, Degner JF, Pique-Regi R, Pai AA, Crawford GE, Stephens M, Gilad Y, Pritchard JK (2012). Dissecting the regulatory architecture of gene expression QTLs. Genome Biol.

[b32] Gamazon ER, Ziliak D, Im HK, LaCroix B, Park DS, Cox NJ, Huang RS (2012). Genetic architecture of microRNA expression: implications for the transcriptome and complex traits. Am J Hum Genet.

[b33] Gaulton KJ, Nammo T, Pasquali L, Simon JM, Giresi PG, Fogarty MP, Panhuis TM, Mieczkowski P, Secchi A, Bosco D, Berney T, Montanya E, Mohlke KL, Lieb JD, Ferrer J (2010). A map of open chromatin in human pancreatic islets. Nat Genet.

[b34] Gennarino VA, D’Angelo G, Dharmalingam G, Fernandez S, Russolillo G, Sanges R, Mutarelli M, Belcastro V, Ballabio A, Verde P, Sardiello M, Banfi S (2012). Identification of microRNA-regulated gene networks by expression analysis of target genes. Genome Res.

[b35] Georges M (2011). The long and winding road from correlation to causation. Nat Genet.

[b36] Georges M, Coppieters W, Charlier C (2007). Polymorphic miRNA-mediated gene regulation: contribution to phenotypic variation and disease. Curr Opin Genet Dev.

[b37] Gong J, Tong Y, Zhang HM, Wang K, Hu T, Shan G, Sun J, Guo AY (2012). Genome-wide identification of SNPs in microRNA genes and the SNP effects on microRNA target binding and biogenesis. Hum Mutat.

[b38] Griffiths-Jones S, Saini HK, van Dongen S, Enright AJ (2008). miRBase: tools for microRNA genomics. Nucleic Acids Res.

[b39] Haas U, Sczakiel G, Laufer SD (2012). MicroRNA-mediated regulation of gene expression is affected by disease-associated SNPs within the 3’-UTR via altered RNA structure. RNA Biol.

[b40] Halvorsen M, Martin JS, Broadaway S, Laederach A (2010). Disease-associated mutations that alter the RNA structural ensemble. PLoS Genet.

[b41] Hariharan M, Scaria V, Brahmachari SK (2009). dbSMR: a novel resource of genome-wide SNPs affecting microRNA mediated regulation. BMC Bioinformatics.

[b42] Harismendy O, Notani D, Song X, Rahim NG, Tanasa B, Heintzman N, Ren B, Fu XD, Topol EJ, Rosenfeld MG, Frazer KA (2011). 9p21 DNA variants associated with coronary artery disease impair interferon-gamma signalling response. Nature.

[b43] Hiard S, Charlier C, Coppieters W, Georges M, Baurain D (2010). Patrocles: a database of polymorphic miRNA-mediated gene regulation in vertebrates. Nucleic Acids Res.

[b44] Hindorff LA, Sethupathy P, Junkins HA, Ramos EM, Mehta JP, Collins FS, Manolio TA (2009). Potential etiologic and functional implications of genome-wide association loci for human diseases and traits. Proc Natl Acad Sci USA.

[b45] Hu Z, Bruno AE (2011). The influence of 3′UTRs on microRNA function inferred from human SNP data. Comp Funct Genomics.

[b46] Huang S, Wu S, Ding J, Lin J, Wei L, Gu J, He X (2010). MicroRNA-181a modulates gene expression of zinc finger family members by directly targeting their coding regions. Nucleic Acids Res.

[b47] Jabs WJ, Logering BA, Gerke P, Kreft B, Wolber EM, Klinger MH, Fricke L, Steinhoff J (2003). The kidney as a second site of human C-reactive protein formation in vivo. Eur J Immunol.

[b48] Jackson AL, Levin AA (2012). Developing microRNA therapeutics: approaching the unique complexities. Nucleic Acid Ther.

[b49] Kim HK, Prokunina-Olsson L, Chanock SJ (2012). Common genetic variants in mir-1206 (8q24.2) and mir-612 (11q13.3) affect biogenesis of mature mirna forms. PLoS ONE.

[b50] Kim J, Bartel DP (2009). Allelic imbalance sequencing reveals that single-nucleotide polymorphisms frequently alter microRNA-directed repression. Nat Biotechnol.

[b51] Kim VN (2005). MicroRNA biogenesis: coordinated cropping and dicing. Nat Rev Mol Cell Biol.

[b52] Kim VN, Han J, Siomi MC (2009). Biogenesis of small RNAs in animals. Nat Rev Mol Cell Biol.

[b53] Kozomara A, Griffiths-Jones S (2011). miRBase: integrating microRNA annotation and deep-sequencing data. Nucleic Acids Res.

[b54] Landi D, Barale R, Gemignani F, Landi S (2011). Prediction of the biological effect of polymorphisms within microRNA binding sites. Methods Mol Biol.

[b55] Lee BK, Bhinge AA, Iyer VR (2011). Wide-ranging functions of E2F4 in transcriptional activation and repression revealed by genome-wide analysis. Nucleic Acids Res.

[b56] Lee Y, Kim M, Han J, Yeom KH, Lee S, Baek SH, Kim VN (2004). MicroRNA genes are transcribed by RNA polymerase II. EMBO J.

[b57] Li J, Liu Y, Xin X, Kim TS, Cabeza EA, Ren J, Nielsen R, Wrana JL, Zhang Z (2012a). Evidence for positive selection on a number of MicroRNA regulatory interactions during recent human evolution. PLoS Genet.

[b58] Li MJ, Wang P, Liu X, Lim EL, Wang Z, Yeager M, Wong MP, Sham PC, Chanock SJ, Wang J (2012b). GWASdb: a database for human genetic variants identified by genome-wide association studies. Nucleic Acids Res.

[b59] Li Y, Nie Y, Cao J, Tu S, Lin Y, Du Y, Li Y (2012c). G-A variant in miR-200c binding site of EFNA1 alters susceptibility to gastric cancer. Mol Carcinog.

[b60] Liang H, Landweber LF (2007). Hypothesis: RNA editing of microRNA target sites in humans?. RNA.

[b61] Liang H, Li WH (2009). Lowly expressed human microRNA genes evolve rapidly. Mol Biol Evol.

[b62] Lin Y, Nie Y, Zhao J, Chen X, Ye M, Li Y, Du Y, Cao J, Shen B, Li Y (2012). Genetic polymorphism at miR-181a binding site contributes to gastric cancer susceptibility. Carcinogenesis.

[b63] Lu J, Clark AG (2012). Impact of microRNA regulation on variation in human gene expression. Genome Res.

[b64] Lucks JB, Mortimer SA, Trapnell C, Luo S, Aviran S, Schroth GP, Pachter L, Doudna JA, Arkin AP (2011). Multiplexed RNA structure characterization with selective 2′-hydroxyl acylation analyzed by primer extension sequencing (SHAPE-Seq). Proc Natl Acad Sci USA.

[b65] Luo X, Yang W, Ye DQ, Cui H, Zhang Y, Hirankarn N, Qian X, Tang Y, Lau YL, de Vries N, Tak PP, Tsao BP, Shen N (2011). A functional variant in microRNA-146a promoter modulates its expression and confers disease risk for systemic lupus erythematosus. PLoS Genet.

[b66] Marson A, Levine SS, Cole MF, Frampton GM, Brambrink T, Johnstone S, Guenther MG, Johnston WK, Wernig M, Newman J, Calabrese JM, Dennis LM (2008). Connecting microRNA genes to the core transcriptional regulatory circuitry of embryonic stem cells. Cell.

[b67] Maurano MT, Humbert R, Rynes E, Thurman RE, Haugen E, Wang H, Reynolds AP, Sandstrom R, Qu H, Brody J, Shafer A, Neri F (2012). Systematic localization of common disease-associated variation in regulatory DNA. Science.

[b68] Mishra PJ, Humeniuk R, Mishra PJ, Longo-Sorbello GS, Banerjee D, Bertino JR (2007). A miR-24 microRNA binding-site polymorphism in dihydrofolate reductase gene leads to methotrexate resistance. Proc Natl Acad Sci USA.

[b69] Mishra PJ, Mishra PJ, Banerjee D, Bertino JR (2008). MiRSNPs or MiR-polymorphisms, new players in microRNA mediated regulation of the cell: introducing microRNA pharmacogenomics. Cell Cycle.

[b70] Mitchell PS, Parkin RK, Kroh EM, Fritz BR, Wyman SK, Pogosova-Agadjanyan EL, Peterson A, Noteboom J, O’Briant KC, Allen A, Lin DW, Urban N (2008). Circulating microRNAs as stable blood-based markers for cancer detection. Proc Natl Acad Sci USA.

[b71] Musunuru K, Strong A, Frank-Kamenetsky M, Lee NE, Ahfeldt T, Sachs KV, Li X, Li H, Kuperwasser N, Ruda VM, Pirruccello JP, Muchmore B (2010). From noncoding variant to phenotype via SORT1 at the 1p13 cholesterol locus. Nature.

[b72] Nica AC, Montgomery SB, Dimas AS, Stranger BE, Beazley C, Barroso I, Dermitzakis ET (2010). Candidate causal regulatory effects by integration of expression QTLs with complex trait genetic associations. PLoS Genet.

[b73] Nicolae DL, Gamazon E, Zhang W, Duan S, Dolan ME, Cox NJ (2010). Trait-associated SNPs are more likely to be eQTLs: annotation to enhance discovery from GWAS. PLoS Genet.

[b74] Nishinakamura R, Takasato M (2005). Essential roles of sall1 in kidney development. Kidney Int.

[b75] Onat A, Can G, Hergenc G (2008). Serum C-reactive protein is an independent risk factor predicting cardiometabolic risk. Metab Clin Exp.

[b76] Ozsolak F, Poling LL, Wang Z, Liu H, Liu XS, Roeder RG, Zhang X, Song JS, Fisher DE (2008). Chromatin structure analyses identify miRNA promoters. Genes Dev.

[b77] Pomerantz MM, Ahmadiyeh N, Jia L, Herman P, Verzi MP, Doddapaneni H, Beckwith CA, Chan JA, Hills A, Davis M, Yao K, Kehoe SM (2009). The 8q24 cancer risk variant rs6983267 shows long-range interaction with MYC in colorectal cancer. Nat Genet.

[b78] Potkin SG, Macciardi F, Guffanti G, Fallon JH, Wang Q, Turner JA, Lakatos A, Miles MF, Lander A, Vawter MP, Xie X (2010). Identifying gene regulatory networks in schizophrenia. NeuroImage.

[b79] Quach H, Barreiro LB, Laval G, Zidane N, Patin E, Kidd KK, Kidd JR, Bouchier C, Veuille M, Antoniewski C, Quintana-Murci L (2009). Signatures of purifying and local positive selection in human miRNAs. Am J Hum Genet.

[b80] Richardson K, Lai CQ, Parnell LD, Lee YC, Ordovas JM (2011). A genome-wide survey for SNPs altering microRNA seed sites identifies functional candidates in GWAS. BMC Genomics.

[b81] Richardson K, Nettleton JA, Rotllan N, Tanaka T, Smith CE, Lai CQ, Parnell LD, Lee YC, Lahti J, Lemaitre RN, Manichaikul A, Keller M (2012). Gain-of-function lipoprotein lipase variant rs13702 modulates lipid traits through disruption of a microrna-410 seed site. Am J Hum Genet.

[b82] Ritz J, Martin JS, Laederach A (2012). Evaluating our ability to predict the structural disruption of RNA by SNPs. BMC Genomics 13 Suppl.

[b83] Roush S, Slack FJ (2008). The let-7 family of microRNAs. Trends Cell Biol.

[b84] Saini HK, Enright AJ, Griffiths-Jones S (2008). Annotation of mammalian primary microRNAs. BMC Genomics.

[b85] Saunders MA, Liang H, Li WH (2007). Human polymorphism at microRNAs and microRNA target sites. Proc Natl Acad Sci USA.

[b86] Schaub MA, Boyle AP, Kundaje A, Batzoglou S, Snyder M (2012). Linking disease associations with regulatory information in the human genome. Genome Res.

[b87] Schmeier S, Schaefer U, MacPherson CR, Bajic VB (2011). dPORE-miRNA: polymorphic regulation of microRNA genes. PLoS ONE.

[b88] Schnall-Levin M, Rissland OS, Johnston WK, Perrimon N, Bartel DP, Berger B (2011). Unusually effective microRNA targeting within repeat-rich coding regions of mammalian mRNAs. Genome Res.

[b89] Senanayake U, Das S, Vesely P, Alzoughbi W, Frohlich LF, Chowdhury P, Leuschner I, Hoefler G, Guertl B (2012). miR-192, miR-194, miR-215, miR-200c and miR-141 are downregulated and their common target ACVR2B is strongly expressed in renal childhood neoplasms. Carcinogenesis.

[b90] Sethupathy P, Collins FS (2008). MicroRNA target site polymorphisms and human disease. Trends Genet.

[b91] Shen LX, Basilion JP, Stanton VP (1999). Single-nucleotide polymorphisms can cause different structural folds of mRNA. Proc Natl Acad Sci USA.

[b92] Shin C, Nam JW, Farh KK, Chiang HR, Shkumatava A, Bartel DP (2010). Expanding the microRNA targeting code: functional sites with centered pairing. Mol Cell.

[b93] Stitzel ML, Sethupathy P, Pearson DS, Chines PS, Song L, Erdos MR, Welch R, Parker SC, Boyle AP, Scott LJ, Margulies EH, Boehnke M, Furey TS, Crawford GE, Collins FS (2010). Global epigenomic analysis of primary human pancreatic islets provides insights into type 2 diabetes susceptibility loci. Cell Metab.

[b94] Stuveling EM, Hillege HL, Bakker SJ, Gans RO, De Jong PE, De Zeeuw D (2003). C-reactive protein is associated with renal function abnormalities in a non-diabetic population. Kidney Int.

[b95] Sullivan PF (2012). Puzzling over schizophrenia: schizophrenia as a pathway disease. Nat Med.

[b96] Thomas LF, Saito T, Saetrom P (2011). Inferring causative variants in microRNA target sites. Nucleic Acids Res.

[b97] van Rooij E, Purcell AL, Levin AA (2012). Developing microRNA therapeutics. Circ Res.

[b98] Volinia S, Galasso M, Costinean S, Tagliavini L, Gamberoni G, Drusco A, Marchesini J, Mascellani N, Sana ME, Abu Jarour R, Desponts C, Teitell M (2010). Reprogramming of miRNA networks in cancer and leukemia. Genome Res.

[b99] Ziebarth JD, Bhattacharya A, Chen A, Cui Y (2012). PolymiRTS Database 2.0: linking polymorphisms in microRNA target sites with human diseases and complex traits. Nucleic Acids Res.

[b100] Zorc M, Skok DJ, Godnic I, Calin GA, Horvat S, Jiang Z, Dovc P, Kunej T (2012). Catalog of microRNA seed polymorphisms in vertebrates. PLoS ONE.

